# Iron Availability in Tissue Microenvironment: The Key Role of Ferroportin

**DOI:** 10.3390/ijms22062986

**Published:** 2021-03-15

**Authors:** Elena Gammella, Margherita Correnti, Gaetano Cairo, Stefania Recalcati

**Affiliations:** Department of Biomedical Sciences for Health, University of Milan, 20133 Milano, Italy; elena.gammella@unimi.it (E.G.); margherita.correnti@unimi.it (M.C.)

**Keywords:** ferroportin, iron, macrophages, microenvironment

## Abstract

Body iron levels are regulated by hepcidin, a liver-derived peptide that exerts its function by controlling the presence of ferroportin (FPN), the sole cellular iron exporter, on the cell surface. Hepcidin binding leads to FPN internalization and degradation, thereby inhibiting iron release, in particular from iron-absorbing duodenal cells and macrophages involved in iron recycling. Disruption in this regulatory mechanism results in a variety of disorders associated with iron-deficiency or overload. In recent years, increasing evidence has emerged to indicate that, in addition to its role in systemic iron metabolism, FPN may play an important function in local iron control, such that its dysregulation may lead to tissue damage despite unaltered systemic iron homeostasis. In this review, we focus on recent discoveries to discuss the role of FPN-mediated iron export in the microenvironment under both physiological and pathological conditions.

## 1. Introduction

Nearly all forms of life require iron for key physiological and developmental processes, such as oxygen transport, energy production and cell proliferation [1]. Only a limited number of microorganisms appear to be able to thrive without taking advantage of the capacity of iron to exchange electrons [2]. Indeed, a bioinformatics approach showed that about 400 (2%) of human genes code for iron proteins which bind the metal either directly or as heme groups and Fe–S clusters [3]. On the other hand, ferrous iron is quite toxic due to its propensity to transform, via the Fenton and Haber–Weiss reactions, relatively mild reactive oxygen species (ROS), like superoxide and hydrogen peroxide, in the highly reactive hydroxyl radical, which can damage membrane lipids, proteins and DNA and cause cell death and tissue damage [4]. That iron represents a potential danger for every organism is also indicated by the fact that yeast cells preferentially produce energy through fermentation, which is energetically less efficient than respiration but does not require the numerous heme and Fe–S iron proteins involved in oxidative metabolism, thereby limiting exposure to iron. In this context, it is not surprising that progressive intracellular ferrous iron accumulation during aging leads to ferroptosis-mediated cell death and lifespan in *C. elegans* [5]. Evidence is accumulating to indicate that iron is involved also in human aging, as shown by recent studies reporting that genes for heme metabolism are related to lifespan and healthspan [6] and indicating that higher systemic iron status may reduce life expectancy [7].

The control of iron balance is particularly crucial in the context of infection/inflammation, in which pathogens use multiple mechanisms to acquire iron, whereas the host sequesters iron and starves them of this essential metal. The universality of this response is highlighted by the appreciation that this competition for iron occurs also in plants, as iron sequestration is important for plant immune responses [8].

Macrophages, which handle a large proportion of the daily iron turnover, play a key role in the regulation of iron availability in mammals [9]. Macrophages seem to withstand iron with less damage than other cells, possibly because in response to heme loading they are able to reprogram their bioenergetic metabolism by decreasing oxidative phosphorylation and shifting to the pentose phosphate pathway that provides NADPH [10]. This mechanism decreases mitochondrial ROS production and, at the same time, NADPH facilitates the maintenance of redox homeostasis, e.g., by functioning as cofactor of the antioxidant protein heme oxygenase 1. Another pathway involved in heme tolerance may be represented by a mechanism leading to crystallization of excess heme into hemozoin. In fact, it has been shown that macrophages lacking the heme exporter HRG1 retain high levels of heme into erythrophagosomes; however, they tolerate this heme burden by forming hemozoin biocrystals, which is the same detoxification stratagem used by blood-feeding parasites to avoid heme toxicity [11].

## 2. Control of Iron Homeostasis

Since iron is a double-edged sword, sophisticated mechanisms have evolved to maintain iron balance both at the systemic and cellular levels. Cellular iron homeostasis is regulated through multiple control mechanisms, but the iron regulatory proteins (IRP)-dependent post-translational regulation, which controls the expression of proteins involved in iron uptake (transferrin receptor, TfR1 and DMT1), storage (H and L ferritin subunits), release (ferroportin (FPN)) and utilization (e.g., eALAS), is probably the most relevant [12]. Body iron is mainly regulated by the hepcidin/FPN axis [13]; hepcidin is a liver-derived peptide that exerts its function by controlling the presence on the cell surface of FPN, which could be considered the sole cellular iron exporter, although it has been recently shown that prominin2 promotes the formation of ferritin-containing multivesicular bodies and exosomes that transport iron out of the cell, thereby facilitating ferroptosis resistance [14].

Hepcidin binding inhibits iron release by triggering FPN internalization and degradation [13] and occluding its cavity [15]. As such, the hepcidin/FPN axis is the main regulator of intestinal iron uptake from dietary sources and iron release by splenic and hepatic macrophages involved in iron recycling from red blood cell breakdown [13]. Interestingly, an additional source of iron for the circulation has been described: erythroblasts also, despite their high iron consumption, return iron to the blood circulation through FPN (transcribed from the alternative mRNA not subject to IRP control) and significantly contribute to serum iron levels [16].

In these erythroid precursors, FPN is highly expressed and exports the iron presumably made available by both TfR1-mediated uptake and heme degradation; the latter could be catalyzed by heme oxygenase, which is expressed in erythroblasts [17], and/or derived by hemoglobin auto-oxidation [18].

Disruption in these regulatory mechanisms results in a variety of disorders associated with iron deficiency or overload. Notably, iron deficiency anemia, which affects two billion people worldwide, represents the most common nutritional disorder.

FPN seems to be localized in a strategic position at the crossroads of these pathways. Over recent years, increasing evidence has emerged to indicate that, in addition to its role in systemic iron metabolism, FPN may play an important function in local iron control, such that its dysregulation may lead to tissue damage despite unaltered systemic iron homeostasis. In this review, we discuss the importance of local iron availability in the microenvironment as an essential factor to maintain tissue homeostasis or repair. Particular attention is paid to iron recycling by macrophages, which is essential for erythropoiesis and may also be relevant for iron redistribution to neighboring cells at the local tissue level [9].

## 3. Ferroportin

FPN (SLC40A1), a member of the large solute carrier gene family, is a trans-membrane protein expressed ubiquitously, primarily in macrophages and duodenal and hepatic cells, as described above. The finding that mice with global FPN inactivation died during embryonic development confirmed its role as the only (or major) iron exporter (reviewed in [19]).

The structure of FPN and the molecular mechanism of iron export are not entirely clear, but an accepted model indicates that membrane FPN extrudes ferrous iron, as suggested by the facilitation of iron export exerted by ferroxidases, which allow successive ferric iron binding by transferrin [19]. Interestingly, recent structural studies of human and primate FPN detailed the structure of the molecule, including the metal binding sites. Moreover, analysis of the interaction between hepcidin and FPN showed preferential targeting of FPN molecules actively engaged in iron export, whereas in the absence of iron the binding affinity of hepcidin remained well below its physiological concentration [20,21]. More information about the structure of FPN and other members of its class of transporters is available in another article of this Special Issue, Ferroportin and Ceruloplasmin: Structure and Function in Health and Disease [22].

**Figure 1:**
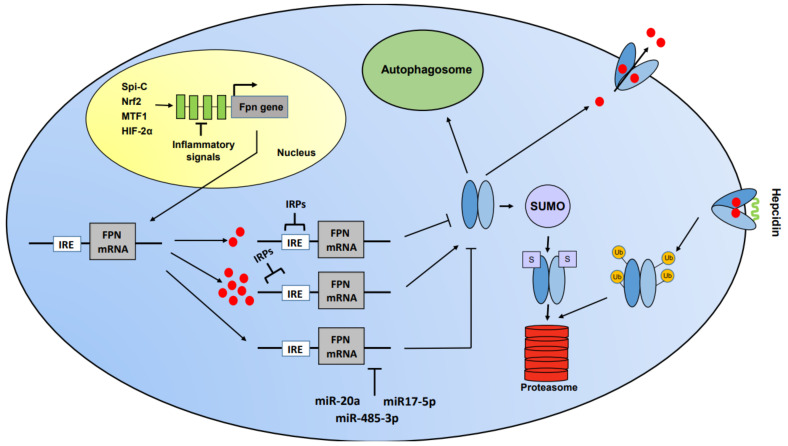
Multilevel regulation of ferroportin (FPN) expression. Transcriptional control: the transcription factors Spic, Nrf2, MTF1 and HIF-2α activate ferroportin transcription whereas still unknown factors responding to inflammatory signals inhibit FPN transcription. Post-transcriptional control: under conditions of iron deficiency, active iron regulatory proteins (IRPs) bind the iron regulatory element (IRE) in the 5′ end of FPN mRNA, thereby blocking its translation. The 3′ end of FPN mRNA is targeted by miRNAs which prevent FPN expression. Post-translational level: in the cytoplasm, FPN can be targeted to the autophagosome or, upon sumoylation, to the proteasome. At the cell surface, where FPN releases iron in the extracellular space or the bloodstream, hepcidin blocks iron transport through the pore and results in the internalization, ubiquitination and proteasomal degradation of FPN.

Probably because of its non-redundant role, the synthesis and activity of FPN are subjected to multilevel control (Figure 1). FPN expression is regulated at the transcriptional level by a number of stimuli, such as exposure to heme, hypoxia or inflammatory mediators. Transcriptional induction by heme and lack of oxygen depend on nuclear factor erythroid 2-related factor 2 (NRF2)- and hypoxia-inducible factor (HIF)-responsive elements, respectively, that have been found and characterized in the FPN promoter region [23,24]. The role of NRF2 has been recently confirmed by a report showing that FPN transcription is downregulated by sirtuin 2, a member of the sirtuin family involved in several cellular processes, including tumorigenesis, metabolism and inflammation [25]. Sirtuin 2 deacetylates NRF2, thereby decreasing its nuclear levels; the resulting lower transcription of FPN leads to an increase in cellular iron. FPN is also highly expressed in macrophages expressing the transcription factor Spi-C, which is necessary for the development of splenic macrophages involved in red blood cells removal [26], although in this context FPN may be also induced by exposure to heme and iron rather than by direct Spi-C-dependent transcriptional induction. Recently, it has been demonstrated that the DNA damage response serine/threonine kinase ATM (mutated in ataxia–telangiectasia) increases FPN expression by enhancing the nuclear translocation of metal-regulatory transcription factor 1 (MTF1) [27], which was previously found to trigger FPN expression [28].

Less well-characterized transcriptional mechanisms responding to Toll-like receptor (TLR) activation control FPN downmodulation in inflammation, a stratagem preventing pathogen access to iron [29]. An intriguing observation has been recently reported about the fine modulation of FPN expression in inflammation. Alam and colleagues demonstrated that in response to TLR/NF-kB signaling, Spi-C induces FPN transcription, thereby promoting iron efflux from macrophages [30]. This mechanism, which is also involved in the reduction of the inflammatory response, may prevent excessive iron retention in macrophages during the resolution phase of inflammation when local iron deficiency in the microenvironment may jeopardy tissue repair [31]. Additionally, epigenetic regulation of FPN has been recently described in primary cultures of endothelial cells of the rat blood–brain barrier [32].

At the post-transcriptional level, FPN synthesis is controlled through the IRP/iron regulatory element (IRE) system [12]. In fact, FPN mRNA contains an iron regulatory element in its 5′ untranslated region that is recognized by the IRPs. Under conditions of iron deficiency, IRPs actively bind to the IRE and prevent FPN mRNA translation, thereby impairing iron export. Conversely, when iron levels are high, IRP binding activity is decreased, leading to efficient translation of FPN mRNA and ultimately decreasing iron content within the cell. Interestingly, cell specific use of alternative splicing of FPN mRNA that bypasses the IRE allows FPN expression and elevated iron export also under conditions of iron scarcity in intestinal and erythroid cells [16]. An additional mechanism of FPN post-transcriptional regulation is through microRNAs. In fact, miR-485-3p and miR17-5p are induced under iron deficiency and target the 3′ UTR of FPN mRNA, thus preventing iron export [33]. Moreover, it has been shown that the levels of miR-20a, which binds to highly conserved target sites in FPN 3’ UTR, are inversely correlated to FPN expression in lung cancer [34]. A final regulation of FPN expression is at the post-translational level and mainly depends on the interaction with hepcidin (see above). However, with regard to post-translational control, it has been recently demonstrated that FPN protein turnover is affected also by cellular iron levels, as hepcidin-dependent degradation is delayed in iron deficiency [35]. Moreover, FPN subcellular trafficking has been suggested to depend on the small ubiquitin-like modifier (SUMO), as a sumoylation-defective mutant was found to be constitutively active, leading to low intracellular iron content [36]. Furthermore, the demonstration that FPN is an autophagic substrate highlighted a novel post-translational control of FPN expression. Indeed, autophagy-dependent FPN degradation may represent an additional regulatory mechanism to block iron release, an effect contributing to ferroptosis and enhancing ferroptosis-mediated tumor suppression [37].

How strong and important are the contributions of each of these pathways to the control of FPN expression? The mechanism based on the interaction between hepcidin and FPN, which leads, for example, to infection-dependent hypoferremia in inflammation, is considered to be the major regulator of iron release, as shown by the relatively small hypoferremia in hepcidin knockout mice [38]. However, it has been demonstrated that the decrease of FPN mRNA can account for the hypoferremia triggered by bacterial lipopolysaccharides [39]. Moreover, a mathematical model of systemic iron regulation showed that the transcriptional control of FPN expression may represent the major determinant of decreased iron levels in the circulation under inflammatory conditions [40]. It is possible to conclude that both molecular mechanisms concur to amplify the hypoferremia response in inflammatory settings. More generally, specific pathways might play a prevalent role depending on the pathophysiological context.

### 3.1. Lessons from Clinical Studies and Mouse Models

Mutations in FPN lead to disorders of iron metabolism characterized by substantially diverse phenotypes [41]. Gain-of-function mutations causing hepcidin resistance are characterized by high transferrin saturation, elevated serum ferritin and parenchymal iron overload, which are features not dissimilar from those typically found in patients with HFE-linked hemochromatosis. Conversely, ferroportin disease (FD) is an autosomal dominant hereditary iron loading disorder in which heterozygote loss-of-function mutations of the gene coding for FPN severely affect iron export, thus leading to progressive and preferential iron trapping in tissue macrophages and high serum ferritin but normal/low circulating iron [42]. The mild clinical presentation of FD patients may suggest that FPN-mediated iron export is not clinically relevant, but the heterogeneous and limited clinical information available for this rare disease may result in under-appreciation of the auxiliary pathological role of macrophage iron accumulation, for example, in fibrogenesis and carcinogenesis. On the other hand, the evaluation of patients with FPN-related diseases has been relevant for our knowledge of the structure, function and regulation of FPN; the definition of its interaction with hepcidin was also improved [15]. The presence of iron overload in FD patients with loss-of-function mutations and hence reduced duodenal iron absorption indicates the existence of differences of FPN production and activity between enterocytes and macrophages, which could be related to FPN transcriptional and translational regulation, assistance in iron export by different oxidases (membrane-bound hephaestin for enterocytes and circulating ceruloplasmin for macrophages) or to the tenfold difference in iron trafficking between these two cell types [43]. FPN could also play a role in HFE-linked genetic hemochromatosis. Recently, it has been shown that HFE deletion targeted to myeloid cells positively regulated FPN and prevented iron accumulation in macrophages of old mice [44]. These results, which confirm previous work indicating that iron export from macrophages is inhibited by HFE [45], are in line with the findings reported in a study preceding FPN identification and characterization, in which we found that macrophages of hemochromatosis patients were paradoxically iron deficient despite body iron overload [46].

With regard to animal experimental models, mice with cell-specific FPN knockout or knockin of hepcidin-unresponsive mutants of FPN have been instrumental for increasing our understanding of the role of FPN in regulating iron levels (see examples below). Indeed, total body inactivation of FPN as well as cell-targeted inactivation of FPN in specific cells resulted in local and/or systemic iron deficiency, sometimes leading to anemia. In addition, the use of cell-specific hepcidin inactivation made possible the characterization of autocrine/paracrine mechanisms controlling iron export based on hepcidin secretion by non-hepatic cells and interaction with FPN on the surface of the same cells.

## 4. Role of Ferroportin-Mediated Modulation of Iron Availability in Tissue Microenvironment

FPN is mainly expressed in the cells involved in systemic iron homeostasis reported above, but it is also expressed ubiquitously and, especially, by tissue resident macrophages. Therefore, it may play an important role in local iron control and its dysregulation can lead to tissue damage despite unaltered systemic iron homeostasis. We provide here some recent and significant examples, which may be representative of the role of FPN in local iron regulation. Tissue specific regulation of iron availability in other organs has been recently covered [47].

### 4.1. Heart

Iron control is essential for cardiac function and can be affected by local or systemic defects. Indeed, loss of FPN-mediated intestinal iron absorption may result in iron deficiency and cardiac damage (see Section 4.2). On the other hand, cardiomyocytes are particularly vulnerable to iron overload.

The analysis of mice with cardiomyocyte-targeted ablation of the FPN coding gene has generated conflicting results: one study showed iron accumulation within cardiomyocytes and consequent heart dysfunction leading to fatal dilated cardiomyopathy despite an unaltered systemic iron level and downmodulation of TfR1-mediated iron uptake, thereby showing that FPN-dependent iron release is necessary to maintain cardiac iron homeostasis [48]. Conversely, another short report failed to find significant consequences [49]. This discrepancy could be possibly related to the dissimilar deletion strategies and the use of different promoters driving Cre recombinase. The role of the hepcidin/FPN axis in the heart was also shown by the demonstration that hepcidin produced locally by cardiomyocytes has relevant autocrine effects and participates in the autonomous regulation of iron in cardiomyocytes, independently from systemic iron regulation. In fact, contrary to systemic hepcidin, cardiac hepcidin increases under conditions of iron deficiency to preserve the cellular iron. Both cardiac-specific hepcidin knockout and knockin of the hepcidin-resistant FPN variant resulted in tissue-specific iron depletion and heart failure, thus showing once again that iron deficiency can likewise result in heart damage [50]. Remarkably, the pathophysiology of cardiac damage was different according to whether systemic or local control of iron metabolism was affected. Therefore, experiments involving heart-specific manipulation of the hepcidin/FPN axis showed the importance of cell-autonomous control of iron balance to maintain normal cardiac functions [51].

### 4.2. Intestine

FPN activity in the intestine plays a well-known key role in body iron homeostasis [13]. Given the importance of duodenal cells in iron absorption, FPN ablation exclusively in intestinal epithelial cells may affect systemic iron homeostasis; indeed, disruption of intestinal iron absorption caused by inducible and cell specific deletion of FPN resulted in progressive iron deficiency anemia and heart damage characterized by massive cardiac hypertrophy [52]. However, recent evidence indicated additional important functions of gut FPN. It has been demonstrated that in intestinal inflammation, increased FPN degradation promoted mucosal healing by preventing iron access to tissue-infiltrating pathogens of the microbiota [53]. Interestingly, this response occurred in the absence of changes in circulating hepcidin levels and hepatic hepcidin synthesis. Instead, FPN was targeted by hepcidin produced locally by dendritic cells activated by microbiota-derived signals; in turn, diminished FPN-mediated iron release from macrophages changed the gut microbiota, facilitating the growth of bacteria that were less iron-dependent and more beneficial for mucosal healing. Another demonstration of the cell-autonomous role of FPN was provided by a study showing that, when hepcidin is low, FPN-mediated iron export from duodenal cells leads to impaired activity of iron-dependent prolyl hydroxylase enzymes. This leads to the stabilization of intestinal HIF-2α, which in turn activates specific target genes involved in iron absorption from the intestinal lumen (Dcytb, DMT1) and basolateral export (FPN). By this mechanism, therefore, FPN export activity contributes to maintaining elevated transcript levels in a feedforward circuit aimed at maximizing iron efflux [54].

### 4.3. Lung

Chronic lung diseases—in particular chronic obstructive pulmonary disease (COPD), which encompasses chronic bronchitis, small airways disease and emphysema, predominantly associated with bacterial infection—are among the leading causes of death worldwide, accounting for about 10 million deaths every year. The pathogenesis of COPD remains poorly understood, but involves inflammatory responses of the lung, most commonly triggered by cigarette smoke. Accordingly, a strong body of evidence implicates a prominent role for alveolar macrophages as drivers of chronic inflammation in COPD. Abnormal iron metabolism has been proposed as a candidate factor involved in COPD susceptibility, with evidence supporting a role for both iron deficiency (and related anemia) and overload. Pulmonary dysfunction has been associated with altered iron balance [55] and disruption of the hepcidin/FPN axis resulted in iron overload in specific pulmonary cell types, in particular alveolar macrophages, leading to compromised lung function [56]. In addition, it has been reported that murine alveolar macrophages exposed to smoke have higher FPN and elevated iron levels [57]. Notably, since alveolar macrophages from smokers display increased expression of hepcidin as well, these results suggest the occurrence of FPN upregulation at the transcriptional level. In polarized cells, FPN is usually expressed basolaterally, but it has been reported that in pulmonary epithelial cells is expressed on the apical membrane [58]. Given this peculiar localization, which would imply the release of iron to the airway lumen, FPN may play a key role in iron detoxification in the lungs. On the other hand, this activity could also be counterproductive; in fact, high FPN-mediated iron release has been suggested to play a role in respiratory infections caused by bacterial species, such as *S. pneumoniae*, which require iron for survival and growth [59].

Although the connection between iron dysregulation and lung disease, such as COPD, is undeniable, additional research is needed to elucidate the underlying molecular mechanisms, particularly the role of FPN-dependent iron export.

### 4.4. Placenta-Fetus

The growing fetus requires a considerable amount of iron, which is supplied by the mother. The syncytiotrophoblast is a polarized cell layer in the placenta which mediates this iron transport. Accordingly, in this tissue, FPN is highly expressed on the basolateral membrane where it exports to the fetal vasculature maternal iron acquired at the apical surface through TfR1. In this context, FPN plays a non-redundant role, as indicated by the demonstration that, when its export activity was preserved in only the placenta, the embryonic lethality observed in mice with global FPN knockout was rescued [60]. The essential role of FPN in placental iron trafficking is also indicated by human studies showing that placental FPN expression increased with gestation age [61]. Understanding the control of iron trafficking from the mother to the fetus was hampered by the existence of a complex interplay of several players: maternal, placental and fetal. However, the clever use of cell-specific genetic manipulation of iron-related genes recently led to considerable progress in these settings.

The suppression of maternal hepcidin normally occurring during pregnancy is essential for iron transfer to the embryo and prevention of adverse outcomes. Under iron-limited conditions, FPN-dependent iron export to fetal circulation is limited to the amount not needed to satisfy the request of the syncytiotrophoblast, a mechanism largely controlled by maternal hepcidin and hence also influenced by inflammatory conditions which induce hepcidin [62]. This selfish placental behavior in the face of possible fetal iron deficiency is probably aimed at maintaining placental iron levels sufficient for essential functions, e.g., mitochondrial respiration, thereby protecting the fetus from the more severe consequences of general placental failure [63]. Fetal hepcidin could control the rate of placental iron transfer to the fetus by regulating the levels of placental FPN which, being expressed on the basolateral side of syncytiotrophoblasts, is accessible only to fetal hepcidin. However, it has been shown that hepcidin produced by fetal liver controls blood fetal iron stores through a mechanism that does not involve significant effects on placental FPN [64]. Elucidation of the molecular determinants underlying the control of placental iron trafficking may be relevant to prevent the deleterious effects of iron deficiency for both mother and fetus.

### 4.5. Liver

A recent study showed that hepatocyte-specific expression of NCOA4, the protein that directs ferritin to autophagosomes and lysososomes, is required for erythropoiesis [65]. Obviously, in order to be used by the erythron, iron must exit the liver; therefore, it can be inferred that FPN expression in hepatocytes is also necessary for a prompt iron mobilization in response to increased demand. Among liver cells, FPN activity is not important only in hepatocytes; in fact, an interesting study demonstrated that FPN is expressed also by hepatic stellate cells and is regulated in a paracrine-like way by hepcidin. Production of hepcidin correlates with activation of these cells, which are major contributors to liver fibrosis [66].

### 4.6. Hair

The development of alopecia has been associated with iron deficiency in humans [67] and was also found in mice with altered expression of proteins of iron metabolism [68,69,70], although in these settings it has not been possible to distinguish the relative contribution of systemic iron deficiency/anemia vs. local iron availability. We have recently shown that genetic deletion of FPN in macrophages resulted in skin lesions and transient alopecia due to impaired proliferation in rapidly growing cells of the hair follicle [31]. Iron retention in resident macrophages starved the neighboring hair follicle cells of iron and hence inhibited their growth. Hair loss was not related to systemic iron deficiency or anemia, thus indicating the necessity of local FPN-mediated iron release from macrophages. These findings revealed that FPN-dependent iron efflux plays a largely underestimated role in the macrophage trophic function in skin homeostasis.

## 5. Role of Ferroportin-Mediated Modulation of Local Iron Availability in Pathological Settings

FPN-mediated modulation of iron availability in the tissue microenvironment, independent from systemic regulation, may be relevant in a number of pathophysiological situations. Here, we focus on recent discoveries and report some relevant cases.

### 5.1. Infection-Inflammation

In the perennial fight between microbial pathogens and host, the competition for iron is crucial and macrophages are the major players in so-called innate nutritional immunity [71]. Therefore, FPN expression is particularly relevant not only in erythrophagocytosing macrophages but also in macrophages involved in innate immunity. In fact, studies of human [72] and mouse [73] macrophages showed that key iron-related proteins are differently regulated according to macrophage polarization. In particular, FPN levels are low in M1 proinflammatory cells and high in M2 macrophages endowed with anti-inflammatory and tissue-remodeling functions, in line with the iron-sequestering and iron-recycling phenotypes, respectively, of these two polarized populations [74]. While polarized macrophages show distinct level of expression of iron proteins, differences in intracellular iron levels do not always influence macrophage polarization; for example, iron accumulation caused by targeted deletion of FPN did not alter the expression of M1 and M2 markers [74]. On the other hand, other studies associated high iron levels with the M1 phenotype (reviewed in [75]), whereas acute iron deprivation strongly affected macrophage energy metabolism and dampened inflammation [76]. The different amount and forms of iron (heme vs. non-heme) can possibly account for these conflicting results.

FPN is also a major player in the iron-immunity crosstalk; interference with iron acquisition by pathogens is a critical component of the immune response and is a widespread mechanism, present also in plants [8]. Several studies have shown the importance of macrophage FPN in infections, with distinct effects on pathogen growth according to whether microbes are extracellular or intracellular [19,77,78]. Notably, an additional mechanism of host nutritional immunity has been recently documented: removal of FPN from the phagosomal membrane during phagocytosis has been identified as a mechanism aimed at preventing the transport of iron from the cytosol into the phagosome lumen where it can be used by phagocytosed microbes [79].

Recently, it has been shown that the crosstalk between host and microbes does not occur only in the context of infectious diseases. In fact, metabolites produced by *Lactobacillus* species in the gut microbiota according to intestinal iron content, by inhibiting HIF-2α, impair iron absorption, thereby regulating systemic iron homeostasis [80].

### 5.2. Cancer

Given the requirement of iron for cell growth [81], cancer cells, which are generally characterized by elevated proliferative potential, have a greater metabolic demand for iron than normal cells. In fact, tumor cells avidly bind iron and the expression of high levels of transferrin receptor (TfR1) to internalize transferrin-bound circulating iron was observed long ago (reviewed in [82]). More recently, alternative mechanisms of iron acquisition by tumor cells have been described; lipocalin-2 is a secreted protein that binds iron-loaded siderophores and can thus serve as an iron delivery vehicle upon internalization mediated by its receptor, LCN2R. Indeed, it has been shown that lipocalin-2 has pro-tumorigenic effects in experimental tumor models and its overexpression is associated with decreased survival in patients [83]. A recent study showed that lipocalin-2 also plays a critical role in leptomeningeal metastasis [84]. Another mechanism used by tumors to increase cellular iron levels is to inhibit iron export; impaired FPN-mediated iron egress helps in maintaining the high levels of the metal necessary for the elevated metabolic requirements of growing cancer cells and thus sustains tumorigenesis. Torti’s group first showed that the levels of FPN were reduced in breast cancer cells in comparison to nonmalignant breast epithelial cells and indicated that FPN expression represents a strong and independent predictor of prognosis in breast cancer [85]. Thereafter, a number of reports showed the close relationship between FPN expression and growth and malignancy of various types of cancer [86]. The hepcidin–FPN interaction can also lead to intracellular iron accumulation in tumor cells. Using breast cancer spheroids, it has been shown that IL6 secreted by fibroblasts present in the tumor microenvironment induces the synthesis of hepcidin in breast cancer cells, which in turn leads to FPN degradation [87].

Iron requirements seem particularly evident in cancer stem cells (CSCs, also termed tumor-initiating cells) which, despite constituting a small fraction of tumor cells, exhibit properties—such as unlimited self-renewal, mesenchymal characteristics, drug- and radiation-resistance and the ability to seed metastases—that are important for tumor progression. The alterations of iron homeostasis, including FPN inhibition, that have recently emerged as key factors in cancer growth and progression are present also in CSCs of a number of different tumors [88]. Interestingly, the key role of iron export has been underpinned by the demonstration that FPN overexpression, similarly to iron chelation, suppressed proliferation and decreased expression of stemness markers.

Upregulation of iron import and downregulation of FPN-dependent iron export and iron storage may result in higher iron availability in cancer cells and consequent faster cell growth. However, it should be considered that enough iron must be available in the microenvironment to achieve an efficient uptake, whereas the tumor microenvironment is nutritionally scarce and characterized by limited concentrations of key resources such as oxygen and iron. To cope with these challenging conditions, cancer cells rely on cells in the tumor microenvironment that are essential to tumor growth. Indeed, cancer cells are able to induce the shift of tumor-associated macrophages (TAMs) to a M2-like phenotype, which is characterized by high FPN levels and an “iron donor” phenotype that eventually fosters tumor growth [77]. Not all TAMs are M2-like and thus some of them could subtract iron from tumor cells. However, at least in the context of leptomeningeal metastasis described above, cancer cells are able to induce a redistribution of iron in the tumor microenvironment and outcompete TAMs for iron. In fact, in these settings, TAMs do not (or not only) directly provide cancer cells with iron, but produce cytokines that stimulate lipocalin-2 expression in cancer cells [84].

### 5.3. Wound Healing

Using mice with iron retention in macrophages due to targeted inactivation of FPN, we investigated the role of macrophage iron release in wound healing, a complex process leading to major clinical problems if impaired. Lack of macrophage FPN led to delayed skin wound healing with defective granulation tissue formation and diminished fibroplasia. Iron retention in macrophages had no impact on the inflammatory processes accompanying wound healing, but affected stromal cells proliferation, blood and lymphatic vessels formation, as well as fibrogenesis [31]. In line with these results, it has been demonstrated that FPN downregulation in macrophages impairs skeletal muscle regeneration after injury [89], thus confirming the importance of FPN-mediated iron export to the microenvironment in tissue repair. Therefore, iron released by M2 macrophages should be added to the list of trophic mediators locally produced by macrophages that stimulate tissue homeostasis and repair. However, it should be kept in mind that macrophages fulfil different functions during the wound healing process and their iron metabolism changes accordingly. In fact, iron accumulation in pro-inflammatory M1 macrophages present in the early phases of an acute injury impaired wound healing [90]. In addition to the effect of different inflammatory signals, the amount and forms of iron may also influence the shift from the M1 to the M2 phenotype and the consequent transition from iron storage to iron release.

### 5.4. Atherosclerosis

The hepcidin–FPN axis seems to play a role in the modulation of the inflammation and polarization of macrophages present in atherosclerotic plaques, but the effects are different in the various stages of the long process of atherosclerosis progression from early lesions to advanced plaques. Indeed, in early- and mid-stage plaques, pro-inflammatory M1 macrophages are predominant (due to lipid ingestion) and increased iron retention caused by low FPN activity could result in increased ROS production, higher oxidized LDL uptake and decreased cholesterol efflux, ultimately leading to plaque progression [91]. Information regarding the role of FPN-mediated iron efflux has been also provided by studies using hepcidin manipulation. In fact, a decrease of intracellular iron due to hepcidin deficiency has been associated with a reduction of pro-atherogenic M1 macrophages independently of serum iron or LDL levels [92]. Conversely, hepcidin administration downregulated FPN-dependent iron export, thereby increasing free iron levels and ROS production [93].

Advanced complex lesions are characterized by angiogenesis with formation of fragile vessels and hence intraplaque hemorrhage. In this context, macrophages showing an M2 phenotype have high hemoglobin content derived from engulfed erythrocytes but are not iron-overloaded and produce low amounts of ROS because of their high FPN expression, which appears to be directly regulated by liver X receptor LXRα, a nuclear receptor known to activate the transcription of genes involved in cholesterol efflux [94].

Iron excess could paradoxically favor the activity of the prolyl hydroxylases and HIF-1α degradation, thus counteracting angiogenesis and leading to plaque stabilization [95]. Recently, the availability of a mouse model, obtained by crossing mice lacking ApoE, which spontaneously develop atherosclerosis, with mice with macrophage-specific FPN deficiency, contributed to highlighting the role of FPN-mediated iron export from macrophages. Iron retention in macrophages increased ROS production, inflammation and plaque lipid composition, thereby remarkably accelerating the progression of atherosclerosis. Notably, FPN deficiency in macrophages inhibited the transcription factor LXRα and the expression of its target genes involved in cholesterol efflux, thus promoting foam cell formation and enhancing plaque progression [96].

## 6. Conclusions

In recent years, we have seen tremendous advances in understanding the pathobiology of iron homeostasis, including the role of FPN-mediated iron release. It is increasingly appreciated that these pathologic conditions are related not only to systemic dysregulation, but also impinge on tissue iron balance, mainly dependent on macrophages, which are critical determinants of local iron supply to the microenvironment.

How can these recent insights into the pathogenesis of iron-related diseases be translated into clinical practice? For example, regarding iron supplementation, hitting the right dose that would provide iron without inducing hepcidin and hence inhibiting FPN-mediated iron release from reticuloendothelial and duodenal cells is a difficult challenge. For this reason, the major emerging therapies seem to be aimed at the manipulation of the hepcidin–FPN axis, using hepcidin agonists or FPN inhibitors for iron overload disorders characterized by inappropriate/low hepcidin and hepcidin antagonists to favor iron release; as, for example, in anemia of inflammation. The hepcidin/FPN agonist/antagonist therapy approach can be also envisaged for the management of iron trafficking in tissue microenvironment.

To date, major efforts have been focused on affecting hepcidin, both directly (e.g., minihepcidins, spiegelmer, anti-hepcidin antibodies) and indirectly through the BMP/BMPR pathway (reviewed in [97]). However, direct FPN targeting has also been pursued; for example, a novel oral FPN inhibitor has been reported [98]. Mechanistic details of both iron- and hepcidin-binding sites will facilitate the development of specific treatments for disorders involving FPN-dependent iron export.

## Abbreviations

COPD	Chronic obstructive pulmonary disease
CSC	Cancer stem cells
FD	Ferroportin disease
FPN	Ferroportin
HIF	Hypoxia-inducible factor
IRE	Iron regulatory element
IRP	Iron regulatory protein
MTF1	Metal-regulatory transcription factor 1
NRF2	nuclear factor erythroid 2-related factor 2
ROS	Reactive oxygen species
SUMO	Small ubiquitin-like modifier
TAM	Tumor-associated macrophage
TLR	Toll-like receptor
TfR1	Transferrin receptor
